# An Analysis of Women’s Fitness Demands and Their Influencing Factors in Urban China

**DOI:** 10.3390/healthcare10020187

**Published:** 2022-01-19

**Authors:** Yupeng Mao, Yongsheng Zhu, Fengxin Sun, Changjun Jia, Bing Liu

**Affiliations:** 1 Physical Education Department, Northeastern University, Shenyang 110819, China; 2001276@stu.neu.edu.cn (Y.Z.); 2171435@stu.neu.edu.cn (F.S.); 2071367@stu.neu.edu.cn (C.J.); 2 School of Arts, Beijing Sport University, Beijing 100084, China

**Keywords:** Chinese women, fitness demands, fitness behavior, fitness motivation

## Abstract

The “Healthy China 2030” plan states that it is necessary to formulate and implement physical health intervention plans for special groups, including women. Based on questionnaire data from women in seven Chinese cities, our research analyzed the status quo of women’s fitness, its influencing factors, and the differences in and characteristics of different types of women’s fitness demands from four aspects: demography, fitness motivation, fitness behavior, and fitness demands, so as to provide a reference for the promotion of women’s fitness. A total of 3473 valid samples were completed. The questionnaire included five age groups: there were 146 in the “20–29 years old” group, 829 in the “30–39 years old” group, 1088 in the “40–49 years old” group, 1105 in the “50–59 years old” group and 305 in the “60 years old and above” group. The questionnaire used in this study was a self-made questionnaire. The contents of the questionnaire included age, occupation, educational level, family circumstances, and health status, women’s fitness behavior, fitness motivation and fitness demands. The results show that the current situation of urban women’s fitness in China is characterized by low frequency and short duration of exercise. The internal factors affecting women’s fitness demands include fitness motivation and fitness behavior. The external factors affecting their fitness demands are social environment and family environment. The differences in women’s fitness demands mainly come from women’s occupation, monthly income, and family stage.

## 1. Introduction

Women are the main force of national health: women’s physical health determines the physique of future generations and even of the whole nation [1,2,3,4,5,6]. Implementing physical fitness programs may be a good way to promote the development of national physical health [7]. The “Healthy China 2030” plan states that it is necessary to formulate and implement physical health intervention plans for special groups such as teenagers, women, and the elderly, that there is an urgent need to strengthen scientific guidance and encourage women, the elderly, and professional groups to actively participate in national fitness programs, and that it is of fundamental importance to encourage and support the construction of new fitness places and venues [8].

A study on the age, occupation and family circumstances of middle-income women by questionnaire showed that Chinese middle-income married women account for 12.8% of the total population. Chinese women’s family work and childcare factors reduce the leisure time of married middle-income women and limit their sports participation behavior [9]. Influenced by gender role expectations and social and cultural discipline, Chinese women have different characteristics in fitness project selection. For example, they mainly participate in aerobics and yoga, and do not like strength training or football. Educational background and economic status result in the social stratification of women’s participation in sports. Married women participate in sports less than unmarried women [10,11]. Time allocation is also an important factor in determining whether women have the time and energy to participate in sports [12,13,14]. In recent years, China’s national policy and social service have changed the lifestyle and fitness concept of individuals. Fitness is considered to be an important means to maintain, intervene in, and promote the overall health of individuals [15]. Popular fitness has increased women’s health experience and endowed women with health subjectivity and health choice ability. Meanwhile, women’s fitness awareness has been enhanced, leading to further demands for fitness activities. Therefore, in order to promote women’s health, we should first pay attention to women’s specific fitness demands and provide health professional services for women. Chinese women’s fitness demands refer to the physical and psychological requirements for fitness that women feel in the process of fitness. Hence, this paper puts forward the first hypothesis: Chinese women’s fitness demands are affected by many factors. Different types (age, occupation, family stage, etc.) of women have different family fitness demands [16,17,18,19,20]. For example, women aged 26–35 participate in sports for leisure and in order to accompany their family [21,22,23,24,25]. The top three reasons why women aged 36–45 participate in sports are to strengthen their health [26,27], relieve stress, and accompany their families [28,29,30,31]. Women aged 46–50 mainly participate in sports to strengthen their health, to accompany their families, and to prevent diseases [32,33,34,35,36]. In addition, women’s participation in fitness activities is related to their social environment and gender culture [37]. Some women pay attention to their own fitness, and some pay attention to family fitness. This is related to the family stage and living environment of women. Therefore, this paper puts forward the second hypothesis: In China, women from different socio-cultural and economic contexts have different fitness demands.

Hence, our paper focuses on women’s individual characteristics (age, occupation, education level, health status), family characteristics (family stage, family population, family monthly income), fitness motivations, fitness behaviors, and fitness demands. Based on the data, our paper describes the current situation of women’s fitness in urban China is characterized by low frequency and short duration. The internal factors affecting women’s fitness demands include fitness motivation and fitness behavior. The external factors affecting their fitness demands are their social environment and family environment. The differences in women’s fitness demands mainly come from their occupations, monthly income, and family stage.

## 2. Methods

### 2.1. Design of Questionnaire

We developed a survey design scheme for the current situation of women’s fitness in urban China. Based on the existing theories of fitness demands, three basic dimensions of women’s fitness demands structure are determined. The three basic dimensions are fitness behavior, fitness motivation and fitness demands. Then, we analyzed the main factors contained in the three basic dimensions. Based on the existing relevant literature, the questionnaire items are compiled. The items of the questionnaire are in Questionnaire S1.

### 2.2. Samples

A total of 3473 valid samples were completed. The questionnaire included five age groups, there were 146 in the “20–29 years old” group, 829 in the “30–39 years old” group, 1088 in the “40–49 years old” group, 1105 in the “50–59 years old” group and 305 in the “60 years old and above” group. We conducted a survey in the form of online questionnaire. The samples were recruited in China’s first tier cities (Beijing, Shanghai, Guangzhou, Shenzhen, Shenyang, Zhengzhou and Kunming). Before the investigation, we trained seven investigators of the research group and unified the investigation methods and standards. Seven investigators went to seven survey cities to distribute online questionnaires face-to-face. The purpose of face-to-face distribution was to improve the survey quality. The network questionnaire was selected to facilitate statistics and avoid errors due to manual input. Finally, a total of 3551 samples were completed from 24 March to 23 April 2020, and after deleting 78 questionnaires of participants who did not meet the recruitment conditions, 3473 valid questionnaires were retained.

### 2.3. Research Instrument

**Figure 1:**
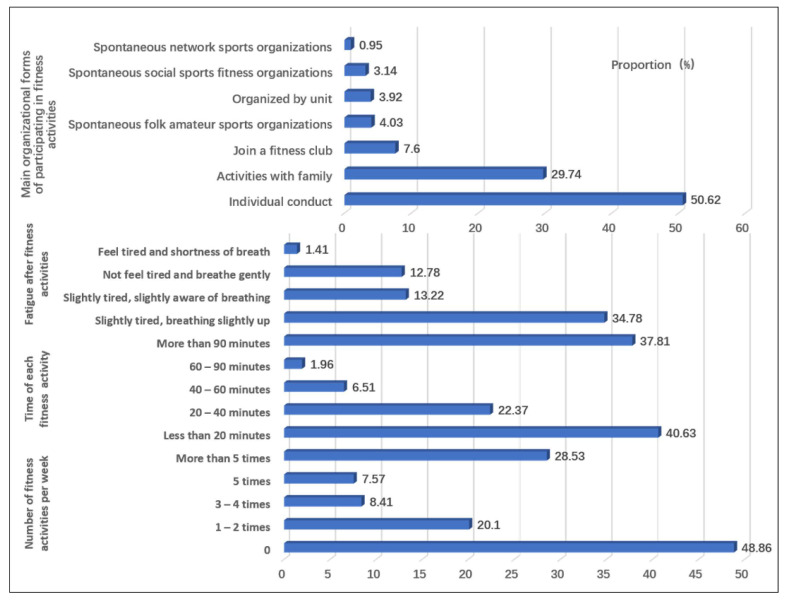
Women’s fitness behaviour and main organizational forms.

**Figure 2:**
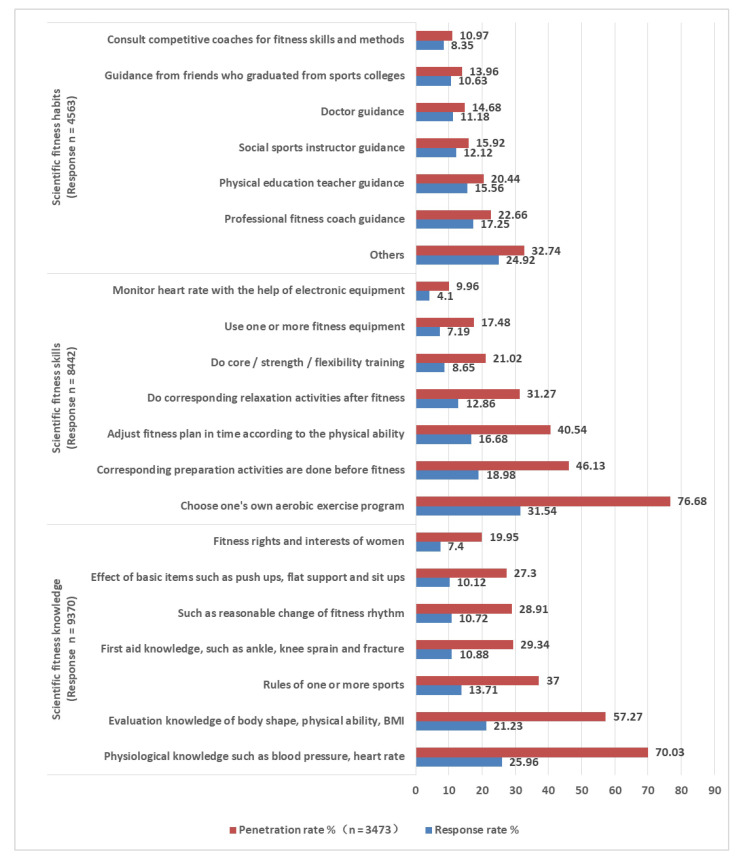
Women’s scientific fitness.

We used a self-made questionnaire to investigate the current situation of women’s fitness in urban China. The questionnaire items mainly come from the results of previous studies. We obtained the description of the current situation of women’s fitness in urban China. The questionnaire contains 40 items. The items of the questionnaire included age, occupation, educational level, family circumstances, health status (Supplementary-Table S1), women’s fitness behavior (Figure 1 and Figure 2), fitness motivation (Table 1) and fitness demands (Table 2).

### 2.4. Statistical Method

We conducted statistics and analysis on the data through SPSS-AU and Excel statistical software. Reliability analysis was used to analyze the reliability of the questionnaire (Supplementary-Table S2). Factor analysis was used to analyze the validity of the questionnaire (Supplementary-Table S3). Frequency analysis was used to analyze the current situation of women’s fitness (Figure 1 and Figure 2). Factor analysis and regression analysis were used to analyze the influencing factors of women’s fitness demands (Supplementary-Tables S4–S6). Factor analysis and cluster analysis were used to analyze the fitness demands of different types of women (Supplementary-Table S7). The differences after clustering were tested by nonparametric test, post hoc multiple comparative analysis, cross analysis, and the chi square test (Table 3 and Table 4).

## 3. Results and Analysis

### 3.1. Women’s Fitness Situation in Urban China

Figure 1 shows the weekly frequency, duration of each time, degree of fatigue and form of participation. In terms of weekly frequency, 84.94% of women participated in fitness activities, but only 36% of them did so regularly. From the perspective of weekly frequency and fatigue, the time spent participating in fitness activities was sufficient, but the exercise intensity was low. From the perspective of the organizational form of the fitness activities, 50.62% of women chose to carry out sports activities alone, while 29.74% chose to participate with their families. This shows that women are more inclined to exercise by themselves.

Figure 2 shows the current situation of women’s scientific fitness. The three multiple-choice topics on scientific fitness were scientific fitness knowledge (health knowledge, sports knowledge), scientific fitness skills (environment utilization skills, fitness method skills), and scientific fitness habits. Scientific fitness knowledge is women’s scientific understanding of fitness. Scientific fitness skills are special skills that women need to master and use in fitness. Scientific fitness habits are ways for women to develop in fitness. In terms of scientific fitness knowledge, the number of people who understood “physiological knowledge such as blood pressure and heart rate” and “physical quality evaluation knowledge such as body shape and physical ability” was 70.03% and 57.27%, respectively. Their understanding of other knowledge was less than 40%. In terms of scientific fitness skills, the number of women who “choose their own aerobic sport” was 76.68%. This may be because women like to choose fitness activities, they are interested in. In terms of scientific fitness habits, the number of women choosing each item was no more than 35%. This shows that researchers should pay attention to women’s fitness guidance.

Women’s fitness motivation is the internal driving force for women to stimulate and maintain fitness and make action-oriented fitness. As shown in Table 1, the proportion of women with strong overall fitness motivation is higher than that of those with weak motivation. This shows that most women have strong fitness motivation. The top five strongest motives include “strengthening physique and promote health”, “improve exercise ability”, “losing weight and shaping”, “please body and mind and relieve pressure”, “improve children’s physical quality “. and “improve family happiness”. The selection proportion of each item is more than 90%. This shows that women hope to promote physical and mental health through physical exercise, use scientific methods to shape and lose weight, and improve external beauty.

Women’s fitness demands are the time, place, environment, etc that women lack in their fitness practice. As shown in Table 2, from the perspective of fitness demands, the proportion of “communication and guidance of fitness knowledge and skills”, “scientific and convenient physique monitoring”, “publicity of women’s fitness”, and “supporting policies and service systems for women’s fitness rights and interests” is higher than 80%. The proportion of “women’s fitness venues, places, and equipment” and “more leisure time” is about 75%. The proportion of “economic condition support” and “fitness performance and competition activities” is about 60%. The proportion of “family sport series activities” is only 35.62%. This shows that women generally need scientific fitness knowledge, skills, physical fitness monitoring, women’s sports policies, and exclusive sports venues, facilities, and free time. Compared with the proportion of the top three demands, the proportion of the bottom three is significantly less, this may be related to women’s individual and family background.

### 3.2. Influencing Factors of Chinese Urban Women’s Fitness Demands

Factor analysis and cluster analysis were used to analyze the fitness demands of different types of women. Five factors are extracted from the scale, they are self-realization fitness motivation, family needs fitness motivation, family support environment demand, public policy demand and social support environment demand (Table S7). Self-realization fitness motivation is the fitness motivation produced by women to realize their own fitness demands. Family needs fitness motivation is the fitness motivation produced by women to meet the fitness demands of family members. Family support environment demand is the demand that women need family members or family environment support in the implementation of fitness behavior. Public policy demand is the demand that women need the support of national policy and fitness service system in the implementation of fitness behavior. Social support environment demand is the demand that women need support such as venues, equipment and women’s fitness publicity in the implementation of fitness behavior.

There is an interaction between women’s fitness demands and family fitness demands. Our research found that in terms of personal background, older women have a higher demand for a family support environment (Table S4). The family support demands of women in different occupations are diverse. Among them, women’s income and free time are significantly related to the demand for a family support environment. Women with a higher education level have lower demand for a family support environment. Women in good health have a high demand for a family support environment. In terms of family background, married women with children have a high demand for a family support environment. In particular, core families have a high demand for a family fitness environment and family support. The larger the family population, the more a family support environment is needed, and this may be related to economic support and having more leisure time to participate in fitness. In terms of fitness motivation, the stronger the motivation of self-realization, the stronger the demand for a family support environment, while the stronger the motivation of family demand, the weaker the demand for a family support environment. This shows that women’s own fitness consciousness can positively affect their family members’ fitness participation. Families with a good family fitness environment will drive women to participate in fitness.

From the perspective of women’s individual fitness, our research found that older women have a higher demand for a social support environment (Table S5). However, when women’s fitness demands and their family background are combined in the process of fitness, it is found that the older they are, the lower their demand for a social support environment. The reason may be that in the family, women’s independent fitness needs the support, encouragement, and guidance of their social environment. When family members exercise together, they can encourage and promote each other, and the demand for a social support environment is relatively weak. Women with a low education level have a high demand for social support. Women with good health have a higher demand for social support, but the correlation is not significant. In terms of family background, families with a high monthly income have a stronger demand for fitness performances, competitions, and family sports activities. The larger the family population, the higher the demand for social support. In terms of fitness motivation, older women with strong motivations of family need and self-realization have a low demand for social support.

From the perspective of women’s individual fitness, women’s demand for public policy is weak (Table S6). However, when women’s fitness and family background are combined in the process of fitness, their demand increases. In terms of family background, the higher the family population and family monthly income, the lower the demand for public policy. In terms of fitness motivation, the stronger the women’s motivation of self-realization, the stronger their demand for public policy. This shows that after women have the motivation to accompany their families and encourage their families to exercise, their own fitness motivation increases their demand for public policy. Conversely, for women with a strong motivation of family demand, it can be inferred that when they participate in sports activities with family members, they need a family support environment and a social support environment more than they need public policy.

### 3.3. Characteristics of and Differences in Fitness Demands of Different Types of Women

**Figure 3:**
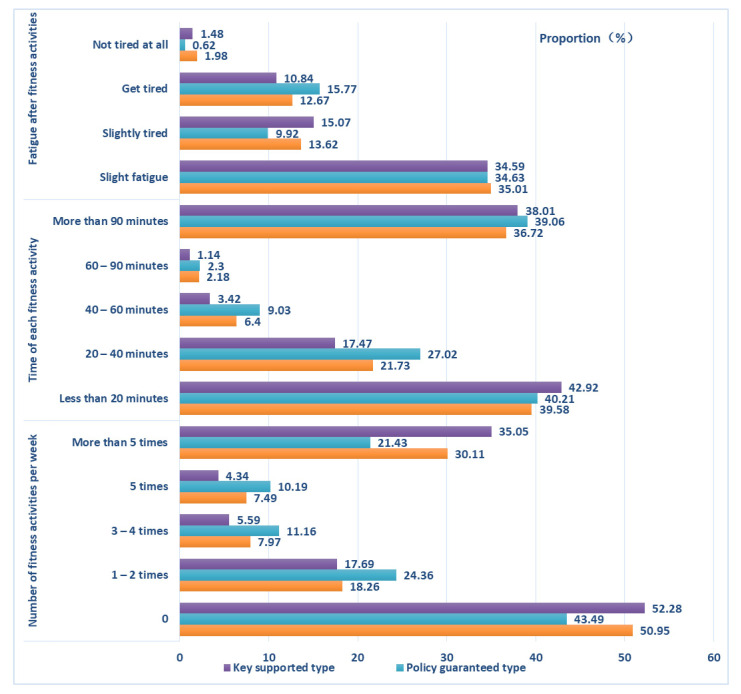
Comparison of fitness activity frequency, durations, and fatigue degree of different types of women.

**Figure 4:**
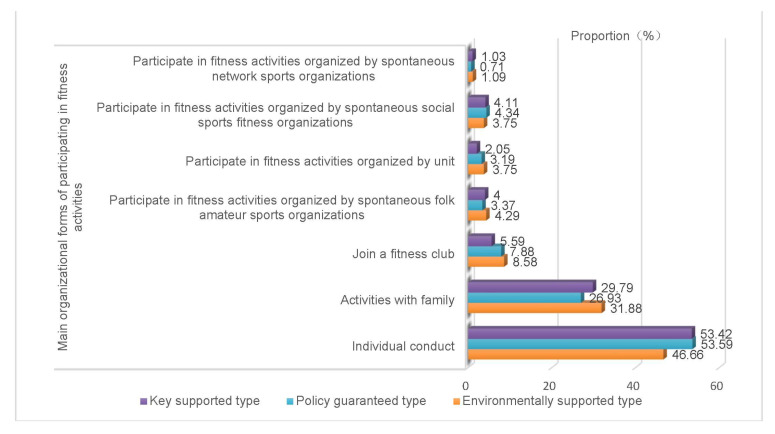
Comparison of main organizational forms of fitness for different types of women.

**Figure 5:**
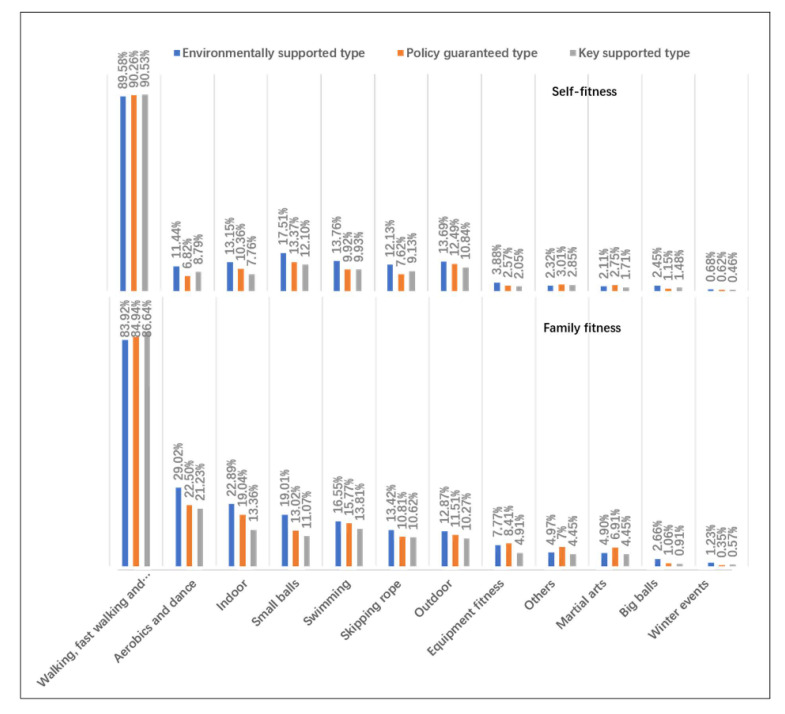
Comparison of fitness style tendency of different types of women facing different participants.

**Figure 6:**
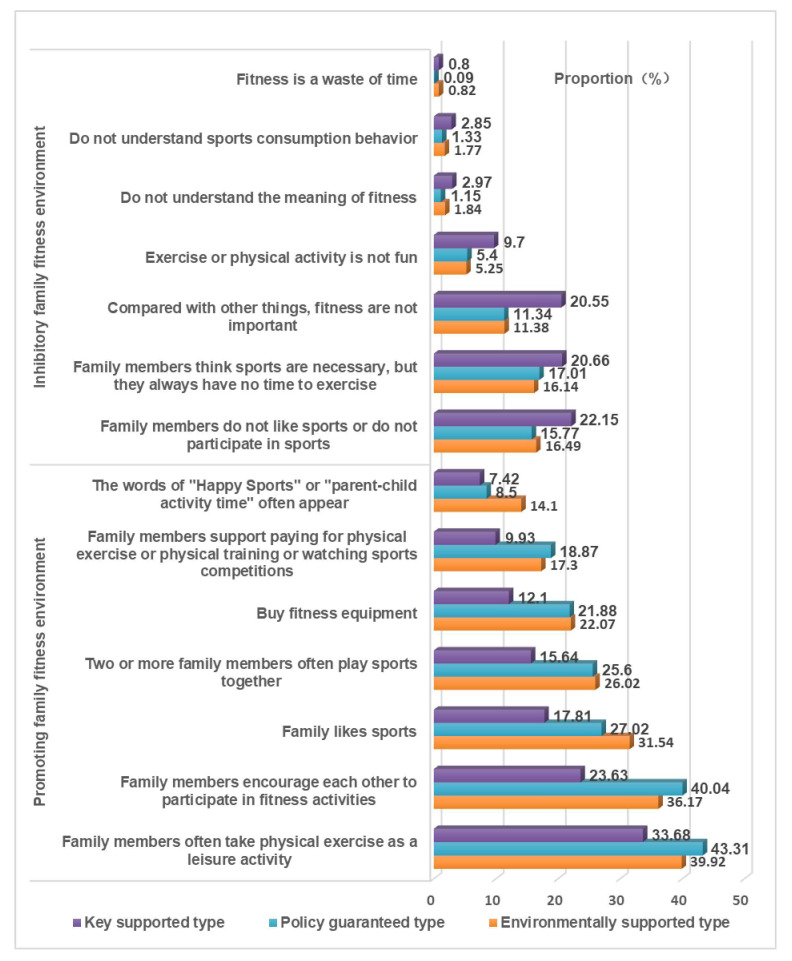
Comparison of family fitness environment for different types of women.

Women’s fitness demands are the time, place, environment, etc that women lack in their fitness practice. According to women’s fitness behavior (Figure 3, Figure 4, Figure 5 and Figure 6), fitness motivation (Table 3), and fitness demand (Table 4), we classify the participants into environmentally supported, policy guaranteed, and key supported groups. The effect size of fitness motivation and fitness demands in Table 3 and Table 4 is at a medium level.

“Environmentally supported group” refers to women who love fitness and have a strong demand for fitness environment, such as family series fitness activities, economic support, etc. Women with a supportive environment have the strongest fitness motivation. Their fitness forms are greatly affected by their families. Their overall fitness behavior is better than that of the other two types. They like to participate in sports activities with their families. When exercising alone, they choose “walking, fast walking, running”, “aerobics and dance”, “indoor”, and “small ball”. When participating in fitness activities with their families, they choose “ball games”, “swimming”, “outdoor”, and “skipping rope”. The family fitness environment of the environmentally supported type is good, and words such as “happy fitness” and “parent–child activity time” often appear. Environmentally supported women have significantly higher fitness demand than the other two types. They need more leisure time and economic support and tend to participate in family sports activities.

“Policy guaranteed group” refers to those who love fitness and hope to obtain a policy guarantee to promote their fitness performance or competition. The family fitness environment of policy guaranteed women is the best of the three types of women. Their number of fitness times per week is the highest, and there are fewer women of this type who never exercise. The fitness time and intensity of each sports activity are better than they are for the other two types. Women in this group like to exercise alone and do physical exercise in parks and gyms. Their family fitness environment is good. When participating in fitness activities with their families, they choose “walking, fast walking, and running”. This type of woman has a high demand for policy guidance, support, and guarantees.

“Key supported group” refers to women who need fitness because of weak fitness motivation, family inhibitory environment and need special attention and help from the society and the government. The fitness motivation of key supported women is weak. This type of woman is mostly born in an inhibitory family fitness environment, and their family fitness consumption expenditure is less. The proportion of this type who exercise more than three times a week is small, and approximately 20% of them never participate in physical fitness activities. Most of them tend to exercise alone and to participate in spontaneous sports activities, such as square dancing and Tai Chi. The demands of key supported women are lower than that of women in the first two groups. Many aging women can be found among the key supported women, which needs the focus of society. Our research found that their fitness motivation needs to be stimulated, they need fitness guarantees, and they need to age healthily.

### 3.4. Discussion

#### 3.4.1. Discussion on the Current Situation and Characteristics of Urban Women’s Fitness in China

The frequency, durations, and fatigue of participating in fitness activities every week can reflect the enthusiasm, habits, and regularity of women who participate in fitness activities. According to the questionnaire survey, women who often participate in fitness are in the minority. Women have enough exercise time to participate in fitness, but their exercise intensity is low. A survey conducted in 2019, Taiwan, China, also showed that women’s sports participation is characterized by insufficient exercise intensity and low participation rate [16]. Combined with women’s fitness demands, the reasons are related to the lack of women’s specialized places for fitness activities, women’s fitness policies [6], and a fitness service system for women. In addition, the survey shows that women, especially married women, lack leisure time. Many studies have shown that women’s lack of leisure time makes them rarely participate in sports and social activities, and indirectly affect their mental health [1,16,32]. Many women bear the responsibilities of raising children, taking care of the elderly, and family work [9,23], and may allocate little time to fitness activities. It can be seen that women’s exclusive fitness services and social division make their fitness participation fail to form a fitness-oriented lifestyle.

#### 3.4.2. Discussion of the Influencing Factors of Urban Women’s Fitness Demands in China

The factors affecting women’s fitness demands include both internal and external environmental factors. Women’s internal factors mainly come from women’s fitness motivation and fitness behavior. If women are short on fitness motivation, their fitness demands will not be engendered [34]. Meanwhile, the external environmental factors affecting women’s fitness demands are mainly reflected in their social and family environment. Many studies have shown that social and family environment affect people’s motivation, especially the motivation and behavior of married women [6,16,26]. Among these, family environment is the main external factor affecting women’s fitness preference or time and opportunity for fitness participation. The survey shows that women’s fitness patterns are different in different family stages. Women who perform housework, support the elderly, and raise children have a strong demand for more leisure time.

#### 3.4.3. Discussion of the Differences in and Characteristics of Urban Women’s Fitness Demands in China

The differences of urban women’s fitness demand in China mainly come from their occupation, monthly income, and family stage [9,10,11]. Women with a high monthly income and stable work environment have a strong demand for women’s specialized places for fitness activities, and married women with children have a strong demand for social public service fitness. The fitness demands of elderly women are weak. According to the analysis of different types of women’s fitness demands in our paper. The fitness motivation of different types of women is consistent in “improving exercise ability”, but all other characteristics are different. Among them, the motivation of women with environmental support is the strongest, followed by policy guarantee, and the motivation of women with key support is weakest. In terms of fitness behavior, our study is consistent with the existing research [12,16,20], that is women’s overall exercise intensity is low. Additionally, there is a general lack of scientific fitness literacy, which urgently needs remedying. Women typically obtain fitness knowledge from the mass media, and their willingness to fitness consumption is low. The fitness behavior of environmentally supported women is better than that of the other two types. In terms of fitness demands, different types of women’s fitness demands show significant differences, and the demands of environmentally supported women are high in the three aspects. Policy guaranteed women have a high demand for public policy. The demands of key supported women across all three aspects are low.

## 4. Conclusions

The current situation of women’s fitness in urban China is characterized by low frequency and short duration. The internal factors affecting women’s fitness demands include fitness motivation and fitness behavior. The external factors affecting their fitness demands are their social environment and family environment. The differences in women’s fitness demands mainly come from their occupations, monthly income, and family stage. According to the research results, we make the following suggestions. First, the society improves women’s fitness cognition and enriches their fitness experience to stimulate their fitness participation behavior. Second, the society and women’s family give full play to the role of sports organizations to meet women’s diverse fitness demands. Third, the government ensures the supply of basic fitness services and promotes women’s fitness participation by relying on various national fitness activities.

In this study, a questionnaire survey was used to explore the fitness demands of women in urban China. However, there is a problem of insufficient scale both in terms of sample size and sample area. Our follow-up studies will expand the sample size and sample area on the basis of this paper. We will deeply explore the influencing factors of women’s fitness demands, the differences between rural women and urban women, the differences between different types of women, and the differences between women and men in fitness demands. In addition, this study lacks in-depth discussion of the influencing factors of women’s fitness demands from the perspective of psychology. For example, the choice tendency of women’s fitness behavior and the strength of fitness motivation are directly affected by women’s psychological factors. Examining the topic of women’s fitness from the perspective of psychology will help researchers collect research data more carefully. Future research should develop our results along the following three aspects. First, the method of stratified sampling should be used to include women in both urban and rural areas in the sample selection range, and the sample size should be expanded. Second, the influencing factors of women’s fitness demands should be studied from the perspective of psychology. This includes studying the relationship between women’s fitness demands and life cycle, and the relationship between women’s fitness demands and emotions. Third, solve the problems of how to effectively supply the fitness demands of different types of women.

## Figures and Tables

**Table 1:** Fitness motivations of the sample population.

Fitness Motivation	Motivational Situation	Frequency	Percentage (%)
Strengthen physique and promote health	Weak Strong	230 3243	6.62 93.38
Satisfy interests and hobbies	Weak Strong	644 2829	18.54 81.46
Pleasure body and mind, relieve stress	Weak Strong	266 3207	7.66 92.34
Improve work efficiency	Weak Strong	451 3022	12.99 87.01
Scientific weight loss and shaping	Weak Strong	253 3220	7.28 92.72
Improve exercise ability	Weak Strong	226 3247	6.51 93.49
Promote emotional communication between husband and wife	Weak Strong	462 3011	13.3 86.7
Improve children’s physical quality	Weak Strong	288 3185	8.29 91.71
Training children to participate in competitive sports	Weak Strong	533 2940	15.35 84.65
Improve family happiness	Weak Strong	424 3049	12.21 87.79

**Table 2:** Fitness demands of the sample population.

Fitness Demand	Demand Situation	Frequency	Percentage (%)
Economic condition support	No Demand	1264 2209	36.4 63.6
More leisure time	No Demand	862 2611	24.82 75.18
Communication and guidance of fitness knowledge and skills	No Demand	533 2940	15.35 84.65
Fitness performance and competition activities	No Demand	1522 1951	43.82 56.18
Family sport series activities	No Demand	1951 1237	56.18 35.62
Scientific and convenient physique monitoring	No Demand	572 2901	16.47 83.53
Women’s fitness venues, places and equipment	No Demand	860 2613	24.76 75.24
Publicity of women’s fitness	No Demand	648 2825	18.66 81.34
Supporting policies and service systems for women’s fitness rights and interests	No Demand	566 2907	16.3 83.7

**Table 3:** Characteristics of fitness motivation of different types of women.

Fitness Motivation	Motivational Situation	Environmentally Supported Type (%)	Policy Guaranteed Type (%)	Key Supported Type (%)	Significance Test χ² (*p*)	φ_c_
Self-realization fitness motivations						
Strengthen physique and promote health	Weak Strong	0.75 99.25	0.89 99.11	23.86 76.14	562.8 (0.000) **	0.40
Satisfy interests and hobbies	Weak Strong	3.47 96.53	15.41 84.59	47.83 52.17	725.5 (0.000) **	0.46
Improve work efficiency	Weak Strong	1.7 98.3	6.82 93.18	39.84 60.16	762.45 (0.000) **	0.47
Pleasure body and mind, relieve stress	Weak Strong	0.14 99.86	1.95 98.05	27.63. 72.37	663.31 (0.000) **	0.44
Scientific weight loss and shaping	Weak Strong	0.54 99.46	1.06 98.94	26.6 73.4	647.23 (0.000) **	0.44
Improve exercise ability	Weak Strong	0.2 99.8	1.24 98.76	23.86 76.14	580.83 (0.000) **	0.41
Family demand fitness motivations						
Promote emotional communication between husband and wife	Weak Strong	2.11 97.89	11.25 88.75	34.7 65.3	511.4 (0.000) **	0.39
Improve children’s physical quality	Weak Strong	0.89 99.11	5.85 94.15	23.86 76.14	393.89 (0.000) **	0.33
Training children to participate in competitive sports	Weak Strong	3.34 96.66	17.27 82.73	32.99 67.01	376.09 (0.000) **	0.33
Improve family happiness	Weak Strong	1.63 98.37	12.13 87.87	30.02 69.98	412.51 (0.000) **	0.33

* *p* < 0.05 ** *p* < 0.01.

**Table 4:** Characteristics of fitness demand of different types of women.

Fitness Demand	Demand Situation	Environmentally Supported Type (%)	Policy Guaranteed Type (%)	Key Supported Type (%)	Significance Test χ² (*p)*	φ_c_
Family support environmental demands						
Economic condition support	No Demand	14.37 85.63	59.34 40.66	43.72 56.28	584.72 (0.000) **	0.41
More leisure time	No Demand	7.56 92.44	40.83 59.17	33.11 66.89	421.7 (0.000) **	0.35
Communication and guidance of fitness knowledge and skills	No Demand	3.81 96.19	17.89 82.11	31.39 68.61	329.51 (0.000) **	0.30
Social support and environmental demand						
Fitness performance and competition activities	No Demand	5.72 94.28	84.32 15.68	55.48 44.52	1666.2 (0.000) **	0.69
Family sport series activities	No Demand	1.98 98.02	69.44 30.56	48.4 51.6	1350.2 (0.000) **	0.62
Public policy demands						
Scientific and convenient physique monitoring	No Demand	3.95 96.05	21.43 78.57	31.05 68.95	322.83 (0.000) **	0.30
Women’s fitness venues, places and equipment	No Demand	5.86 94.14	40.12 59.88	36.64 63.36	490.96 (0.000) **	0.37
Publicity of women’s fitness	No Demand	1.63 98.37	27.9 72.1	35.27 64.73	503.21 (0.000) **	0.37
Supporting policies and service system for women’s fitness rights and interests	No Demand	1.57 98.43	24.18 75.82	30.82 69.18	420.43 (0.000) **	0.35

* *p* < 0.05 ** *p* < 0.01.

## Data Availability

The datasets used and analyzed during the current study are available from the corresponding author on reasonable request.

## Supplementary Materials

The following supporting information can be downloaded at: https://www.mdpi.com/article/10.3390/healthcare10020187/s1, Table S1: Descriptive statistics of sample basic information; Table S2: Reliability of questionnaire; Table S3: Validity of the questionnaire; Table S4: Robust regression results of family support environmental demands; Table S5: Robust regression results of social support environmental demands; Table S6: Robust regression results of public policy demands; Table S7: Comparison results of variance analysis of clustering categories of urban women’s fitness demands.
