# Supplementary material for: An Analysis of Women’s Fitness Demands and Their Influencing Factors in Urban China
Source: Healthcare (Basel). 2022 Jan 19;10(2):187. doi: 10.3390/healthcare10020187 (PMC8872378; doi:10.3390/healthcare10020187)
Supplement: Supplementary file 1 [file healthcare-10-00187-s001.zip › healthcare-1507308-supplementary.pdf]

healthcare MDPI

Table S1 Descriptive statistics of sample basic information

Sample characteristics	Classification criteria	Frequency	Percentage (%)
Age	20-29 years old	146	4.2
	30-39 years old	829	23.87
	40-49 years old	1088	31.33
	50-59 years old	1105	31.82
	60 years old and above	305	8.87
Occupation	Personnel of state organs and civil servants	661	19.03
	Enterprise and trade personnel	610	17.56
	Doctor	493	14.2
	Teacher	828	23.84
	Civilian personnel (scientific researchers, lawyers, secretaries, editors, journalists)	440	12.67
	Others (retired, full-time housewives, engineers, farmers, social workers, unemployed, etc.)	441	12.7
Degree of education	Junior high school and below	21	0.6
	Technical secondary school / high school	100	2.88
	College	305	8.78
	Undergraduate college	1814	52.23
	Graduate student	1233	35.5
Health status	Very good	858	24.7
	Good	1777	51.17
	Generally good	773	22.26
	Not good	65	1.87
Family stage	Married, childless	178	5.13
	Married with children	2936	84.54
	Unmarried	193	5.56
	Widowed	37	1.07
	Divorce	129	3.71
Family population	1 person	90	2.59
	2 persons	465	13.39
	3 persons	1971	56.75
	4 persons	562	16.18
	5 persons	255	7.34
	6 persons	92	2.65
	7 persons and above	38	1.09
Monthly household income	Below 2000 RMB	9	0.26
	2001-4000 RMB	152	4.38
	4001-8000 RMB	737	21.22
	8001-15000 RMB	1197	34.47
	15001-25000 RMB	850	24.47
	25001-50000 RMB	409	11.78
	More than 50001 RMB	119	3.43

Table S2 Reliability of questionnaire

Cronbach reliability analysis			
**Name**	**Total correlation of correction items (CITC)**	**Item deleted α coefficient**	Cronbach α coefficient
Fitness helps to strengthen your physique and promote health.	0.539	0.937	
Fitness can satisfy your interests.	0.611	0.935	
Fitness can delight the body and mind and relieve stress.	0.684	0.934	
Fitness helps to improve work efficiency.	0.681	0.934	
Scientific fitness helps to lose weight and shape scientifically.	0.706	0.934	
Scientific fitness can improve sports ability.	0.727	0.934	
Promote emotional communication between husband and wife in sports activities.	0.693	0.934	

Fitness can encourage children to participate in sports activities and improve their physical quality.	0.722	0.934	
Fitness can improve children's sports skills and interests, and lay a foundation for training children to participate in competitive sports.	0.699	0.934	0.938
Fitness can drive family members to participate in sports activities, make them happy and improve family happiness.	0.733	0.933	
Good economic conditions are needed to support fitness programs.	0.501	0.939	
Need more leisure time.	0.538	0.937	
Need scientific fitness knowledge, skills and other guidance.	0.694	0.934	
Scientific and convenient physique monitoring is needed to understand the health status in time.	0.694	0.934	
Professional and convenient female fitness venues, places and equipment are needed.	0.627	0.935	
Hope to participate in various forms of fitness performances and competitions.	0.608	0.936	
Hope to participate in family sports series.	0.655	0.935	
Hope to strengthen the publicity of women's fitness.	0.717	0.933	
Hope to establish supporting policies and service system for women's fitness rights and interests.	0.699	0.934	
Standardized Cronbach α coefficient：0.943			

Table S3 Validity of the questionnaire

Validity analysis results						
Name	Loading factors					Common degree
	Factor 1	Factor 2	Factor 3	Factor 4	Factor 5	
Fitness helps to strengthen physique and promote health.	0.77	0.089	0.158	0.109	-0.003	0.637
Fitness can satisfy your interests.	0.742	0.237	0.051	0.072	0.261	0.683
Fitness can delight the body and mind and relieve stress.	0.835	0.256	0.154	0.082	0.12	0.807
Fitness helps to improve work efficiency.	0.771	0.339	0.128	0.058	0.182	0.762
Scientific fitness helps to lose weight and shape scientifically.	0.741	0.361	0.224	0.163	0.007	0.757
Scientific fitness can improve sports ability.	0.748	0.383	0.238	0.153	0.017	0.787
Promote emotional communication between husband and wife in sports activities.	0.486	0.607	0.2	0.11	0.173	0.686
Fitness can encourage children to participate in sports activities and improve their physical quality.	0.487	0.715	0.182	0.158	0.066	0.811
Fitness can improve children's sports skills and interests, and lay a foundation for training children to participate in competitive sports.	0.344	0.788	0.16	0.158	0.18	0.822
Fitness can drive family members to participate in sports activities, make them happy and improve family happiness.	0.415	0.777	0.186	0.122	0.175	0.856
Good economic conditions are needed to support fitness programs.	0.071	0.083	0.148	0.815	0.244	0.758
Need more leisure time.	0.115	0.126	0.165	0.828	0.163	0.768
Need scientific fitness knowledge, skills and other guidance.	0.261	0.232	0.553	0.565	-0.008	0.747
Scientific and convenient physique monitoring is needed to understand the health status in time.	0.213	0.207	0.638	0.525	0.034	0.772

Professional and convenient female fitness venues, places and equipment are needed.	0.126	0.076	0.605	0.462	0.293	0.687
Hope to participate in various forms of fitness performances and competitions.	0.144	0.141	0.281	0.311	0.775	0.817
Hope to participate in family sports series.	0.145	0.236	0.377	0.226	0.739	0.816
Hope to strengthen the publicity of women's fitness.	0.244	0.214	0.74	0.113	0.4	0.826
Hope to establish supporting policies and service system for women's fitness rights and interests.	0.232	0.191	0.774	0.129	0.334	0.818
Eigenvalue (before rotation)	9.502	2.59	1.027	0.821	0.677	-
Variance interpretation rate% (before rotation)	50.009%	13.634%	5.405%	4.322%	3.564%	-
Cumulative variance interpretation rate% (before rotation)	50.009%	63.643%	69.048%	73.370%	76.934%	-
Eigenvalue (after rotation)	4.611	2.908	2.806	2.488	1.804	-
Variance interpretation rate% (after rotation)	24.266%	15.306%	14.769%	13.097%	9.496%	-
Cumulative variance interpretation rate% (after rotation)	24.266%	39.572%	54.341%	67.438%	76.934%	-
KMO value	0.941					-
Bartlett's value	51008.57					-
df	171					-
P value	0					-

Table S4 Robust regression results of family support environmental demands

Variables	Robust model			
	Model 1	Model 2	Model 3	Model 4
Age	0.002** (3.076)	0.000** (24.233)	0.000** (-18.402)	0.162 (-1.399)
Occupation	0.000** (-27.891)	0.000** (15.187)	0.000** (37.354)	0.000** (41.416)
Degree of education	0.000** (-16.797)	0.000** (-14.529)	0.000** (-6.053)	0.000** (-6.074)
Health status	0.167 (1.383)	0.003** (2.939)	0.000** (11.493)	0.706 (0.377)
Family stage		0.193 (-1.302)	0.000** (-10.823)	0.000** (-3.99)
Family population		0.000** (-7.306)	0.144 (-1.461)	0.000** (-3.922)
Monthly household income		0.002** (3.112)	0.000** (-6.937)	0.011* (-2.545)
Self-realization fitness			0.000** (4.961)	0.062 (1.863)
Family needs fitness				0.000** (5.36)
Sample size	3473	3473	3473	3473
R^2	-0.111	-0.111	-0.111	-0.111

Adjusted R^2	-0.113	-0.114	-0.114	-0.114
F value	86.953	49.644	43.426	38.590

Note: * p<0.05, ** p<0.01. In parentheses is the value of t value.

Table S5 Robust regression results of social support environmental demands

Variables	Robust model			
	Model 1	Model 2	Model 3	Model 4
Age	0.000**<br>(10.282)	0.000**<br>(-8.074)	0.000**<br>(17.408)	0.000**<br>(-12.238)
Occupation	0.000**<br>(-15.682)	0.000**<br>(-16.758)	0.000**<br>(4.382)	0.000**<br>(24.182)
Degree of education	0.000**<br>(10.316)	0.000**<br>(-6.759)	0.000**<br>(-10.905)	0.005**<br>(2.792)
Health status	0.074<br>(1.784)	0.217<br>(-1.235)	0.000**<br>(-11.585)	0.739<br>(-0.334)
Family stage		0.506<br>(-0.666)	0.135<br>(1.495)	0.018*<br>(2.357)
Family population		0.006**<br>(2.749)	0.271<br>(-1.102)	0.000**<br>(-3.591)
Monthly household income		0.000**<br>(-8.135)	0.000**<br>(-5.281)	0.000**<br>(6.717)
Self-realization fitness			0.000**<br>(13.927)	0.062<br>(1.865)
Family needs fitness				0.128<br>(1.523)
Sample size	3473	3473	3473	3473
R^2	-0.104	-0.104	-0.104	-0.104
Adjusted R^2	-0.106	-0.107	-0.107	-0.107
F value	81.894	46.756	40.900	36.345

Note: * p<0.05, ** p<0.01. In parentheses is the value of t value.

Table S6 Robust regression results of public policy demands

Variables	Robust model			
	Model 1	Model 2	Model 3	Model 4
Age	0.388<br>(-0.863)	0.000**<br>(-10.383)	0.000**<br>(-8.301)	0.000**<br>(19.175)
Occupation	0.000**<br>(7.365)	0.000**<br>(-8.92)	0.000**<br>(-8.998)	0.000**<br>(11.903)
Degree of education	0.000**<br>(-4.888)	0.439<br>(0.774)	0.000**<br>(5.262)	0.000**<br>(-18.715)
Health status	0.95<br>(0.063)	0.389<br>(0.861)	0.000**<br>(-4.961)	0.742<br>(-0.329)
Family stage		0.000**<br>(-16.277)	0.008**<br>(-2.662)	0.000**<br>(7.474)
Family population		0.003**<br>(-2.966)	0.000**<br>(5.54)	0.000**<br>(5.434)
Monthly household income		0.000**<br>(-5.89)	0.002**<br>(3.03)	0.000**<br>(-4.339)
Self-realization fitness			0.000**<br>(3.901)	0.000**<br>(5.661)
Family needs fitness				0.000**<br>(-4.656)
Sample size	3473	3473	3473	3473
R^2	-0.046	-0.046	-0.046	-0.046

Adjusted R^2	-0.047	-0.048	-0.048	-0.048
F value	37.838	21.603	18.897	16.793

Note: * p<0.05, ** p<0.01. In parentheses is the value of t value.

Table S7 Comparison results of variance analysis of clustering categories of urban women's fitness demands

	**Comparison results of variance analysis of cluster categories (mean ± standard deviation)**			F	p
	**Environmentally supported type**	Policy guaranteed type	Key supported type		
**Self-realization fitness motivation**	**0.72±0.45**	-0.97±0.88	0.05±0.74	1930.556	0.000**
Family needs fitness motivation	0.40±0.75	-0.39±1.18	-0.16±0.86	248.134	0.000**
Family support environment demand	0.25±0.61	0.00±1.23	-0.42±1.06	133.094	0.000**
public policy demand	0.22±0.62	0.05±1.18	-0.44±1.12	132.606	0.000**
Social support environment demand	0.35±0.40	0.53±0.63	-1.27±1.01	2087.261	0.000**

* p<0.05 ** p<0.01

Questionnaire S1

Questionnaire on fitness of women and their families

Dear lady:

Hello! In order to collect relevant data on women and their family fitness for scientific research, you are invited to participate in this survey. All the information you provide is strictly confidential and only used for statistical analysis. The opinions released in later scientific research are the summary of a large number of questionnaires and will not reflect your personal specific information. It will take about 5-8 minutes to complete the questionnaire. Thank you for your cooperation!

Part I. (Multiple choice questions, tick "√" before the options you think appropriate)

1. Age

① 20-29 years old ② 30-39 years old ③ 40-49 years old ④ 50-59 years old ⑤ over 60 years old

2. Family stage

① Married, childless

② Married with children

③ Unmarried

④ Widowed

⑤ Divorce

3. Population of your family

① 1 ② 2 ③ 3 ④ 4 ⑤ 5 ⑥ 6 ⑦ 7 and above

4. Family's monthly income

① Below 2000 RMB ② 2001-4000 RMB ③ 4001-8000 RMB ④ 8001-15000 RMB ⑤ 15001-25000 RMB ⑥ 25001-50000 RMB ⑦ More than 50001 RMB

5. Education level

① Junior high school② Technical secondary school or senior high school ③ College ④ Undergraduate ⑤ Master ⑥ Doctor

6. Occupation

① Personnel of state organs and civil servants
② Enterprise and trade personnel
③ Doctor
④ Teacher
⑤ Scientific researchers
⑥ Lawyer
⑦ Secretary
⑧ Edit
⑨ Reporter
⑩ Others (Please fill in)______________

7. Health

① Very good ② Good ③ Average ④ Poor

Part II. (Multiple choice questions, tick "√" before the options you think appropriate)

1. How many times do you participate in sport activities per week?

① 0
② 1-2 times
③ 3-4 times
④ 5 times
⑤ More than 5 times

2. How long do you spend on sport activities?

① Less than 20 minutes
② 20-40 minutes
③ 40-60 minutes
④ 60-90 minutes
⑤ More than 90 minutes

3. What is the general level of fatigue after you participate in sport activities?

① I don't feel tired at all and breathe gently.
② Slightly tired, slightly aware of breathing.
③ Slightly tired, breathing slightly up, but still comfortable.
④ Feel tired, shortness of breath, can talk to people, but prefer not to talk.
⑤ Extremely tired, breathing very hard, exhausted.

4. What is the main organizational form of your participation in sport activities?

① Alone.
② Activities with family.
③ Join a fitness club.
④ Participate in sports activities organized by the unit.
⑤ Participate in sports activities organized by spontaneous social sports fitness organizations.
⑥ Participate in sports activities organized by spontaneous folk amateur sports organizations.
⑦ Participate in sports activities organized by spontaneous network sports organizations.

5. All the main venues where you participate in sport activities. (Multiple choices are allowed)
① Indoor or residential.
② Near the community.
③ Park.
④ Square.
⑤ Work unit.
⑥ Free sports venues.
⑦ Charging gymnasium and fitness place.
6. What do you know about the following contents? (Multiple choices are allowed)
① Physiological knowledge such as blood pressure and heart rate.
② Body shape, physical ability and other physical quality evaluation knowledge.
③ First aid knowledge of common sports injuries such as ankle, knee and waist sprain and fracture.
④ Reasonable scientific fitness knowledge such as reasonable change of fitness rhythm in fitness.
⑤ The effects of basic fitness items such as push-ups, flat support and sit ups.
⑥ The rules of one or more sports.
⑦ Fitness rights and interests of women, children, the elderly and other people.
7. What are your ways to acquire sport knowledge? (Multiple choices are allowed)
① Mass media (Internet, TV, lecture programs, newspapers and periodicals)
② Education (School physical education curriculum, sports school curriculum)
③ Sports activities (Exercises, competitions, performances, etc.)
④ Physical training (Classes, clubs, etc.)
⑤ Individuals (Sports partners, friends, family)
8. Which of the following contents are included in your fitness process? (Multiple choices are allowed)
① Corresponding preparation activities will be done before fitness.
② Choose your own aerobic exercise program.
③ Core / strength / flexibility training.
④ Use one or more fitness equipment.
⑤ After fitness, they will do corresponding relaxation activities.
⑥ During exercise, we will monitor breathing and heart rate at any time with the help of electronic equipment such as sports watch bracelet.
⑦ Adjust the fitness plan in time according to the physical ability at that time.
9. What professional fitness instructions have you received? (Multiple choices)
① Physical education teacher guidance.
② Guidance from friends who graduated from sports colleges.
③ Professional fitness coach guidance.
④ Consult professional competitive sports coaches for fitness skills and methods.
⑤ Social sports instructor guidance.
⑥ Doctor guidance.
⑦ Others. (Please fill in)____________________
10. What are your general fitness methods? (Multiple choices are allowed)
① Walking, fast walking and running.
② Big balls. (Foot, basketball, volleyball, etc.)

③ Small balls. (Table tennis, badminton, tennis, gateball, etc.)
④ Aerobics and dance. (Aerobics, square dance, sports dance, modern dance, etc.)
⑤ Martial Arts. (Taijiquan, sword, fitness Qigong, etc.)
⑥ Rope skipping.
⑦ Swimming.
⑧ Indoor. (Fitness app, fitness video, fitness video games, etc.)
⑨ Outdoor. (Bicycle, mountaineering, rock climbing, fishing, etc.)
⑩ Winter events. (Skating, skiing, ice hockey, curling, etc.)
(11) Fitness equipment. (Dumbbell, high pull-down equipment, leg adduction & Leg abduction equipment, etc.)
(12) Others. (Please fill in)____________________________

11. When you exercise with your family members, which general fitness methods do you choose? (Multiple choices are allowed)

① Walking, fast walking and running.
② Big balls. (Foot, basketball, volleyball, etc.)
③ Small balls. (Table tennis, badminton, tennis, gateball, etc.
④ Aerobics and dance. (Aerobics, square dance, sports dance, modern dance, etc.)
⑤ Martial Arts .(Taijiquan, sword, fitness Qigong, etc.)
⑥ Rope skipping.
⑦ Swimming.
⑧ Indoor. (Fitness app, fitness video, fitness video games, etc.)
⑨ Outdoor. (Bicycle, mountaineering, rock climbing, fishing, etc.)
⑩ Winter events. (Skating, skiing, ice hockey, curling, etc.)
(11) Fitness equipment (Dumbbell, high pull-down equipment, leg adduction & Leg abduction equipment, etc.)
(12) Others. (Please fill in)____________________________

12. Which of the following situations will occur in your family (Option A or Option B)

Option A (Multiple choices)

① My family likes sports.
② My family members often take physical exercise as a leisure activity.
③ My family members support paying for physical exercise or physical training or watching sports competitions.
④ Two or more family members often play sports together.
⑤ My family members encourage each other to participate in sport activities.
⑥ Buy fitness equipment.
⑦ The words of "Happy Sport" or "Parent-child activity time" often appear.

Option B (Multiple choices)

① My family members do not like sports or do not participate in sports.
② Compared with other things, sports are not very important.
③ Exercise or physical activity is not much fun.
④ They can't understand the meaning of fitness.
⑤ They think fitness is a waste of time.

⑥ They think sports are necessary, but they always have no time to exercise.
⑦ Do not understand sports consumption behavior.

13. What is the average monthly fitness consumption of your family?

① Less than 200 RMB
② 201-500 RMB
③ 501-1000 RMB
④ 1001-1500 RMB
⑤ 1501-2000 RMB
⑥ 2001-2500 RMB
⑦ More than 2501 RMB

Part III. (Scale questions)

Please fill in according to your actual situation. The number 1-5 indicates the degree. The larger the value, the higher the degree; Degree 1 means very disagree and degree 5 means very agree. Tick "√" on the number you think appropriate

1. Fitness helps to strengthen physique and promote health.	1	2	3	4	5
2. Fitness can meet interests and hobbies.	1	2	3	4	5
3. Fitness can delight the body and mind and relieve stress.	1	2	3	4	5
4. Fitness helps to improve work efficiency.	1	2	3	4	5
5. Scientific fitness helps to lose weight and shape scientifically.	1	2	3	4	5
6. Scientific fitness can improve sports ability.	1	2	3	4	5
7. Couples participate in sports activities together to promote emotional communication.	1	2	3	4	5
8. Self-fitness can encourage children to participate in sports activities and improve their physical quality.	1	2	3	4	5
9. Self-fitness can improve children's sports skills and interests, and lay a foundation for training children to participate in competitive sports.	1	2	3	4	5
10. Self-fitness can drive family members to participate in sports activities, make them happy and improve family happiness.	1	2	3	4	5
11. We need a good family and social sports environment to improve the participation rate of sports activities.	1	2	3	4	5
12. Need good economic conditions to support fitness programs.	1	2	3	4	5
13. Need more leisure time.	1	2	3	4	5
14. Need scientific fitness knowledge, skills and other guidance.	1	2	3	4	5
15. Scientific and convenient physique monitoring is needed to understand the health status in time.	1	2	3	4	5
16. Professional and convenient women's fitness venues, places and equipment are needed.	1	2	3	4	5
17. They hope to participate in family sports series.	1	2	3	4	5
18. They hope to participate in various forms of fitness performances and competitions.	1	2	3	4	5
19. I hope to increase the publicity of women's fitness.	1	2	3	4	5

20. We hope to establish a supporting policy and service system for women's fitness rights and interests.	1	2	3	4	5

At the end of the questionnaire, thank you again for your cooperation.
Best wishes for you!
